# Ultrasound-assisted size tuning of polyacrylic acid coated magnetic nanoparticle clusters for biomedical applications

**DOI:** 10.1016/j.ultsonch.2026.107876

**Published:** 2026-05-13

**Authors:** Lukas Heinen, Pascal-Raphael Blersch, Constantin Schnell, Karsten Meyer, Roland Nagy, Marcus Halik, Christina Janko, Stefan Lyer, Christoph Alexiou, Rainer Tietze

**Affiliations:** a Department of Otorhinolaryngology – Head and Neck Surgery, Section of Experimental Oncology and Nanomedicine (SEON), Else Kröner Fresenius Foundation Professorship (EKFS), Universitätsklinikum Erlangen, Erlangen, 91054, Bavaria, Germany; b Department of Chemistry and Pharmacy, Inorganic Chemistry, Friedrich-Alexander-Universität Erlangen-Nürnberg, 91058, Bavaria, Germany; c Institute of Applied Quantum Technologies, Friedrich-Alexander-Universität Erlangen-Nürnberg, 91052, Bavaria, Germany; d Organic Materials & Devices, Institute of Polymer Materials, Friedrich-Alexander-Universität Erlangen-Nürnberg, 91058, Bavaria, Germany

**Keywords:** Ultrasound, Magnetic nanoclusters, Size tunability, Cellular loading

## Abstract

The hydrodynamic size of magnetic nanoparticle clusters is a critical determinant of their *in vivo* behaviour and therapeutic efficacy. While alkaline co-precipitation offers a scalable route for polyacrylic acid (PAA) coated superparamagnetic iron oxide nanoparticles (SPIONs), it typically yields polydisperse agglomerates. This work establishes a predictive engineering process using controlled, post-synthesis ultrasound treatment to precisely tune SPION cluster size. Utilising a D-optimal Design of Experiments (DoE) approach, we modelled the influences of sonication parameters on the hydrodynamic diameter, identifying specific energy input as the governing factor for de-agglomeration. The resulting verified regression model (*adj. R*${}^{2}=0.9986$) enables predictable laboratory scale-up across varying volumes (1–10 ml) and concentrations (1–10 mg/ml) while maintaining material integrity. Quantitative magnetic characterisation revealed that ultrasound-induced fragmentation increases the mass-specific susceptibility, which is attributed to the magnetic de-locking of frustrated cores as inter-cluster spacing increases. Crucially, biological evaluations in B16-F10 melanoma cells demonstrate that this ultrasound-assisted size tuning directly influences cellular loading. Cellular iron mass post SPION incubation was found to follow a dual-variable dependency: while iron loading increases with cluster diameter for a fixed core size, it is significantly impacted by the primary core dimensions. SPION clusters with 12 nm cores exhibited a two-fold higher iron loading (8.23 pg Fe/cell) compared to those with 8 nm cores at equivalent hydrodynamic sizes, highlighting the importance of the magnetic payload per cluster. These findings establish a robust framework for engineering SPIONs with tailored dimensions to maximise and predict the magnetic responsiveness of loaded cells, providing a reliable foundation for future applications such as cell tracking, magnetic drug targeting, and hyperthermia.

## Highlights

• Statistical analysis of ultrasound treatment process.
• Simple cluster size tuning by post synthesis ultrasound treatment.
• Cellular load for varying cluster and core sizes.

## Graphical abstract

**Figure:**
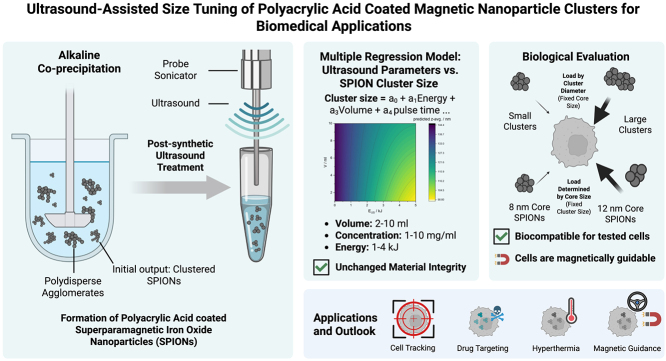

## 1 Introduction

Superparamagnetic iron oxide nanoparticles (SPIONs) have emerged as versatile platforms for numerous biomedical applications, including magnetic drug targeting (MDT) [1], [2], magnetic cell seeding [3], [4], magnetic resonance imaging (MRI) [5], [6], and *in vitro* diagnostics such as critical offset magnetic particle spectroscopy [7], [8] and quantum sensing [9], [10], [11].

Beyond their magnetic properties, the functionality of SPIONs is determined by their surface chemistry, which can be tailored by various coatings like citric acid [4], lauric acid [1], or (3-Aminopropyl) triethoxysilane [8]. A commonly used approach to enhance both stability and functionalisation potential is the application of a polyacrylic acid (PAA) coating [12], [13], [14]. As a biocompatible polymer, PAA is negatively charged under physiological conditions and stabilises nanoparticles by creating strong electrosteric repulsion, which prevents aggregation in suspension [15], [16], [17]. Furthermore, the abundant carboxyl groups on the PAA surface serve as reactive sites for conjugation with molecules such as amino acids [13], drugs [2], [18], or proteins [19].

Next to *in vitro* diagnostics [20], the hydrodynamic size of nanoparticle clusters is a critical parameter for *in vivo* applications, as it dictates both therapeutic efficacy and bloodstream circulation time. For effective biomedical use, nanoparticles or clusters of multiple primary core particles must be in a size range of approximately 10–200 nm, which ensure that they are large enough to avoid renal filtration yet small enough to evade the mononuclear phagocyte system and pass through standard 0.2 μm sterilising filters [21], [22]. While nanoparticle size can be defined during synthesis, methods like thermal decomposition often require significant effort, such as high temperatures, long reaction times, or precisely dosed reactants [23], [24], [25], [26], [27], [28]. In contrast, alkaline co-precipitation offers a simpler, scalable aqueous method, but it typically yields polydisperse particles that form large, uncontrolled clusters [29], [30]. This challenge highlights the need for a straightforward, post-synthesis technique to modulate the cluster size of SPIONs. Ultrasound treatment is an established method for de-agglomerating particle suspensions. The process relies on acoustic cavitation, in which the violent collapse of microscopic bubbles generates intense local shear forces capable of breaking down particle clusters [31], [32], [33], [34]. While sonication has been explored during nanoparticle synthesis for various systems [35], [36], [37], [38], [39], its application for post-synthesis size control of SPION clusters is less developed in detail. Specifically, the field currently lacks a predictive framework that links controllable input parameters to a precise, predictable hydrodynamic output.

In this work, we present a method to precisely tune the cluster size of PAA-coated SPIONs using a controlled post-synthesis ultrasound treatment. The true novelty of our approach lies in the transformation of a common but poorly controlled laboratory procedure into a robust engineering process. Utilising a Design of Experiments (DoE) approach, we develop a verified regression model that quantitatively predicts the final cluster size across varying scales. By evaluating their cellular load and magnetic responsiveness in B16-F10 melanoma cells, we establish a foundational link between predictive process engineering and functional biological outcomes.

## 2 Methods

### 2.1 SPION-PAA synthesis

Polyacrylic acid coated system SPION I was synthesised based on existing methods described in literature [17], [40], [41] but modified. In a typical synthesis, 0.7455 g FeCl${}_{2}$ (Carl Roth, Germany) and 2.0275 g FeCl${}_{3}$ (Carl Roth, Germany) in 40 ml water were stirred at 80 °C under argon at an overhead-stirrer speed of 300 1/min. After 10 min, 15 ml of 25 % ammonia (Carl Roth, Germany) was rapidly added (pH ≈ 11–12). After 15 min, 2.2 g polyacrylic acid (Mw = 2000 Da) (Fisher Scientific GmbH, Germany) in 10 ml water was added and stirred for 2 h. Particles were magnetically collected, re-dispersed in 35 ml water and purified by tangential flow filtration (100 kDa, 750 ml water) and stored at 8 °C until further use.

For system SPION II, precipitation occurred at ambient temperature, followed by PAA addition (after 10 min), heating to 90 °C over 15 min, and stirring at 90 °C for 15 min. Other conditions were kept constant as described for SPION I.

### 2.2 Physical and chemical characterisation

Iron concentration was quantified via microwave-assisted plasma atomic emission spectroscopy (Agilent 4200, Agilent Technologies, USA) at 371.99 nm using external calibration standards (AnalytiChem GmbH, Germany, 0–5000μg/L). SPION samples (20μl) were dissolved with 80μl 65 % nitric acid (Carl Roth, Germany) at 95 °C for 10 min and diluted 5000-fold. Magnetically labelled cells were dried to mass consistency (95 °C), digested in 20μl nitric acid (95 °C, 10 min), and diluted with 980 μl water. Results are presented by the average and the standard deviation of n = 5 independent trials.

Hydrodynamic size (z-average) was determined via dynamic light scattering (DLS, Zetasizer Nano, Malvern Panalytical, UK) in backscattering mode (173°) at 25 °C. Samples (1.2 ml, 50μg Fe/ml) were measured in PMMA cuvettes (SARSTEDT AG & Co. KG, Germany) in triplicate for 120–160 s (averaged 10 s runs). Hydrodynamic size, z-average and hydrodynamic cluster size will be used synonymously, as sizes are determined by means of DLS and at constant iron concentration.

Primary core sizes were measured via transmission electron microscopy (Zeiss EM 912) using SPIONs air-dried on Formvar/Carbon supported copper grids (200 mesh, Merck KGaA, Germany). Images were analysed using a custom Python/OpenCV [42] script involving Gaussian blurring, Otus’s thresholding [43], and morphological opening. A marker-controlled watershed algorithm separated overlapping particles to determine primary size distributions [44]. Statistics of at least 250 events were calculated after IQR outlier removal and plotted using Origin (Version 2023b. OriginLab Corporation, USA).

Volume-specific magnetic susceptibility of SPIONs (1 mg Fe/ml) was measured using a MS2G susceptometer (Bartington Instruments, UK) at 1.3 kHz and 23 °C. Measurements were recorded for 2 s in triplicate, and the mean and standard deviation were calculated.

### 2.3 Tuning hydrodynamic size by ultrasound

Ultrasound was applied using a Bandelin Sonoplus 4100 (MS 73 sonotrode) at an operating frequency of 20 kHz and different Energy inputs, recorded by the device. Samples (V = 1 ml) were sonicated in 2 ml tubes (Eppendorf SE, Germany) aligned 3 cm from the probe tip, while scale-up trials utilised 15 ml tubes aligned at 9 cm. Statistical analysis of predominant input variables and Scale-up was conducted via a custom three level D-optimal screening design of experiments (DoE) by varying iron concentration ($\beta_{\text{Fe}}$), volume (V), power (P), energy (E), and pulsation settings ($t_{\text{on}}$, $t_{\text{off}}$) in 17 individual runs, each executed in triplicates. Results for z-average were fitted to a standard least squares model, comprising the intercept $c_{0}$, linear $x_{i}$, interaction $x_{i}x_{j}$ and quadratic terms $x_{z}^{2}$ scaled by their respective weighting factor c (Eq. (1)).

(1) $$y_{i}=c_{0}+\overset{n}{\underset{i=1}{\sum }}c_{i}x_{i}+\overset{n-1}{\underset{i=1}{\sum }}\overset{n}{\underset{j=1}{\sum }}c_{ij}x_{i}x_{j}+\overset{n}{\underset{z=1}{\sum }}c_{z}x_{z}^{2}$$

Lack-of-fit test was performed and Leave-one-out method was applied as cross-validation. Furthermore, 3 additional experiments were selected to test model validity. To systematically prioritise the process parameters based on their statistical significance, an effect screening was performed using the Logworth statistic. The Logworth value is defined as the negative base-10 logarithm of the p-value:

(2) $$\text{Logworth}=-\log_{10}\left(\text{p-value}\right)$$

All described statistical calculations, as well as the creation of the DoE, were done using JMP Pro 18.0.2 (2025 JMP® JMP Statistical Discovery LLC, USA).

### 2.4 Biological characterisation

B16-F10 melanoma cells (ATCC, USA) were seeded (1.5⋅10${}^{4}$/well, 6-well plates) in DMEM (Gibco®, Life Technologies, UK) containing 10 % fetal bovine serum (FBS) (Sigma-Aldrich, Germany) and 1 % GlutaMAX™ (Gibco®, Life Technologies, UK) and cultured at 37 °C (5 % CO${}_{2}$). After 24 h, sterile filtrated (0.22 μm filter) SPIONs were added to a final concentration of 75μg Fe/ml. H${}_{2}$O and 3 % DMSO served as negative and positive controls, respectively. Following a 24 h incubation, cells were washed with phosphate buffered saline (PBS) to remove residual particles for further analysis. Cells were detached using Trypsin/EDTA solution (PAN-Biotech GmbH, Germany), resuspended in the respective media and centrifuged (220 rcf, 3 min). The supernatant was removed and the cell pellet was resuspended in culture media for subsequent analysis. Cells were analysed in flow cytometry (Gallios, Beckman Coulter, USA). Harvested cells (150μl) were incubated with 200μl of a staining mixture in Ringer’s solutions containing Annexin V-FITC (ImmunoTools, Friesoyte, Germany) (10 pg/ml), Hoechst 33342 (Thermo Fisher Scientific, USA) (10 μg/ml), DilC${}_{1}$(5) (Thermo Fisher Scientific, USA) (2.04 μg/ml), and propidium iodide (Sigma Aldrich, Germany) (PI; 66.6 ng/ml) for 20 min at 4 °C in the dark.

AxV-FITC and PI both were excited at 488 nm; the AxV-FITC fluorescence was recorded on the fluorescence (FL)1 sensor (525/38 nm band pass filter, BP) and the PI fluorescence on the FL3 sensor (620/30 nm BP). DilC${}_{1}$(5) was excited at 638 nm and recorded on an FL6 sensor (675/20 nm BP). Excitation of the Hoechst 33342 fluorescence was at 405 nm and recording on the FL9 sensor (430/40 nm BP). To eliminate fluorescence bleed through electronic compensation was used. Analysis was performed using Kaluza software (Beckman Coulter, USA). All experiments were performed in triplicate and repeated three times independently (n = 3 biological replicates per experiment). The iron mass per cell $\chi_{\text{Fe}}$ was calculated with the measured Fe mass $m_{\text{Fe}}$ by means of MP-AES and the counted cell number (from flow cytometry) $N_{\text{cells}}$:

(3) $$\chi_{\text{Fe}}=\frac{m_{\text{Fe}}}{N_{\text{cells}}}$$

Significances were tested by means of one-way ANOVA and Tukey post-hoc test using Origin (Version 2023b. OriginLab Corporation, USA).

## 3 Results and discussion

### 3.1 Effect of ultrasound treatment on SPION properties

**Fig. 1:**
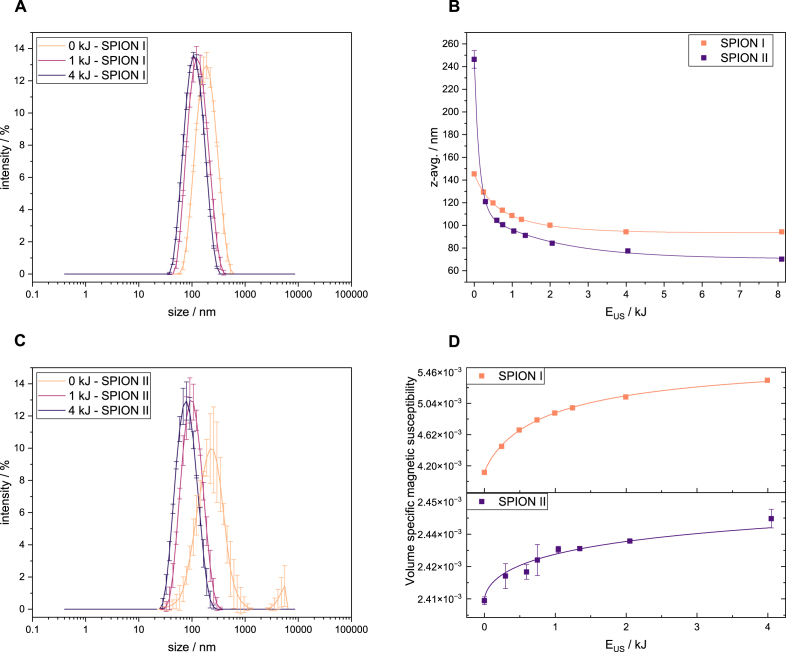
SPION I and SPION II properties as a function of ultrasound energy. A Intensity weighted size distribution of SPION I sonicated with 0, 1 and 4 kJ. B Z-avg. as a function of ultrasound energy. C Intensity weighted size distribution of SPION II sonicated with 0, 1 and 4 kJ. D Volume specific magnetic susceptibility normed to 1 mg Fe per ml as a function of ultrasound energy.

The effect of ultrasonic energy on the hydrodynamic properties of two distinct SPION formulations is presented in Fig. 1. The intensity-weighted size distributions reveal significant differences in the initial agglomeration state and sonication response of SPION I (Fig. 1A) and SPION II (Fig. 1C). Initially, SPION I exhibits a relatively narrow, monomodal size distribution centred around 150 nm. In contrast, the untreated SPION II sample displays a bimodal distribution with a substantial population in the micrometre range, indicating the presence of large agglomerates, arguably due to a higher residual iron content in the solution due to the lower reaction temperature. Residual iron ions will undergo complexation with polyacrylic acid [45], which might result in the formation of larger clusters. Upon application of ultrasonic energy (E${}_{US}$), these large agglomerates are effectively dispersed. For SPION II, even a modest energy input (e.g., 0.5 kJ) eliminates the larger population, resulting in a monomodal distribution. The quantitative analysis of the z-average as a function of ultrasonic energy confirms these observations (Fig. 1B). The z-average of both formulations decreases with increasing energy before converging towards a stable minimum size (z-avg). While SPION II begins with a much larger z-avg (approx. 250 nm) than SPION I (approx. 150 nm), its final z-avg is smaller, converging at around 70 nm compared to around 94 nm for SPION I. This highlights both the high degree of initial agglomeration in SPION II and the high efficiency of the de-agglomeration, meaning that larger clusters break down into multiple smaller clusters. The observed size reduction and plateau is consistent with the fragmentation limit described by Kusters et al. [33], where the internal strength of the primary cluster exceeds the hydrodynamic shear forces generated by cavitation bubbles.

Since intensity weighted size measurements are biased towards larger particles (d${}^{6}$), the derivative of z-avg over ultrasound energy is not necessarily proportional to the change in cluster number. To further analyse that the change in z-avg is not an effect of breaking down only a few larger agglomerates, magnetic properties are compared. The de-agglomeration mechanism is further supported by changes in the volume-specific magnetic susceptibility ($\chi_{V}$), as shown in Fig. 1D. For both SPIONs, $\chi_{V}$ increases with applied ultrasonic energy before reaching a plateau. This behaviour is attributed to inter-particle magnetic interactions. In the initial, agglomerated state, the close proximity and random orientation of nanoparticles lead to complex magnetic dipolar interactions that hinder the alignment of their moments with an external magnetic field, thus lowering the bulk susceptibility per unit volume [46], [47], [48], [49]. As sonication breaks these agglomerates into smaller, well-dispersed clusters, this reduction in inter-particle coupling allows for an enhanced response to the applied field, resulting in an increased measured susceptibility. The inverse relationship and shared convergence point between z-avg and magnetic susceptibility strongly support this interpretation. To ensure that the observed increase in $\chi_{v}$ originates from structural de-agglomeration rather than concentration artefacts or sedimentation, we investigated the dependency of $\chi_{v}$ on the iron mass concentration ($\beta_{\text{Fe}}$) for distinct z-avg. For each hydrodynamic diameter, a strictly linear relationship $\chi_{v}=k\left(\text{z-avg}\right)\cdot \beta_{\text{Fe}}$ was observed (Figure B.3A), confirming the colloidal stability during measurement. Crucially, the mass-normalised susceptibility (the slope k) increased as the cluster size decreased. We attribute this to the reduction of inter particle dipolar interactions. This is supported by the estimated change in cluster number for different hydrodynamic diameters (Figure B.3C). This demonstrates that the observed decrease in z-average is not merely an artefact of breaking a few large agglomerates, but reflects a fundamental shift towards a vastly increased number of smaller, well-dispersed primary clusters.

**Fig. 2:**
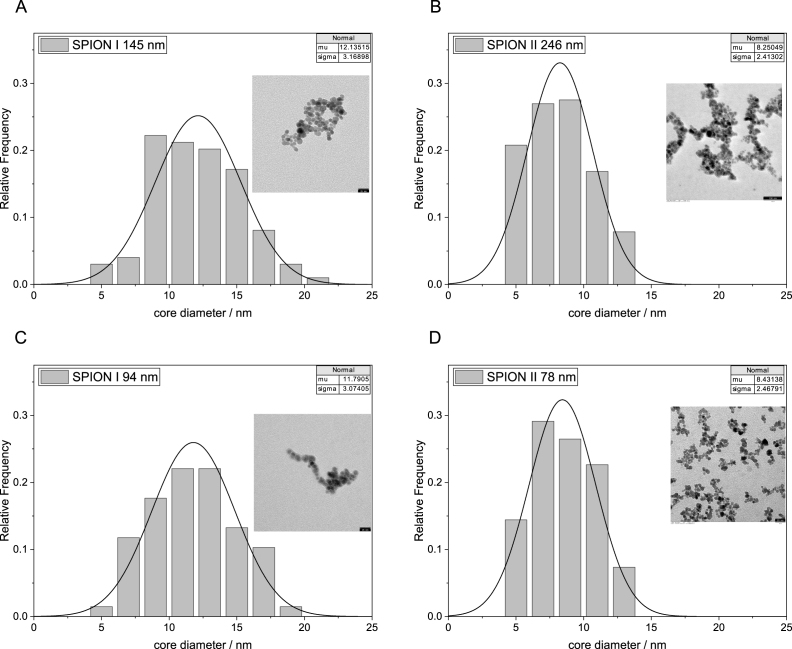
Comparison of core sizes prior and after ultrasound treatment. A Core size distribution of untreated SPION I (z-avg = 145 nm) with one representative TEM image. B Core size distribution of untreated SPION II (z-avg = 245 nm) with one representative TEM image. C Core size distribution of SPION I treated with ultrasound to 94 nm with one representative TEM image. D Core size distribution of SPION II treated with ultrasound to 78 nm with one representative TEM image.

Furthermore, these changes are due to the breakdown of the clusters, not to alterations in the primary particles. Transmission electron microscopy (TEM) images (Fig. 2) confirm that the nanoparticle core sizes remain unaffected by the ultrasound treatment, measuring around 12 nm and 8 nm for SPION I and SPION II respectively.

The reported core sizes are consistent with those co-precipitated particles reported in the literature [12], [14], [17], [50]. Furthermore, the size-adjusted SPION clusters demonstrate excellent colloidal stability, with their hydrodynamic diameters remaining constant for at least 30 days (Figure A.1 C). Also, no alteration in z-avg or the PAA coating could be determined over a cumulative ultrasound treatment up to 20 kJ (Figure A.2). As demonstrated by the temperature profile in Figure C.4, even at higher energy inputs (4 kJ/ml), the bulk temperature remains below 75 °C, well below the thermal degradation onset of PAA as determined by means of thermogravimetric analysis (Figure A.1 D).

The normalised saturation magnetisation reached approximately 91 emu/g and 81 emu/g for untreated SPION I and SPION II, respectively (Table A.1). Post ultrasound treatment with 4 kJ/ml decreases M${}_{s}$ to around 90 emu/g and 74 emu/g for SPION I and SPION II by maintaining their superparamagnetic behaviour. The larger decrease for SPION II can be attributed to a higher surface-initiated oxidation during ultrasonication (Appendix A).

In summary, the combined analysis demonstrates that post-synthesis ultrasound treatment can be used as a robust method for tuning the hydrodynamic cluster size of PAA-coated SPIONs. The process involves the disassembly of larger clusters into smaller and stable clusters. This was verified by corresponding changes in their magnetic susceptibility, while preserving the integrity of the nanoparticle cores. This technique provides a rapid and reliable method of engineering SPION clusters with specific sizes tailored for a range of applications.

### 3.2 Optimisation of ultrasound treatment process

To transform the post-synthesis sonication into a predictable engineering process, a custom D-optimal Design of Experiments (DoE) was employed SPION I. This approach allows for a systematic mapping of the parameter space and the identification of synergistic interactions that govern the final cluster size. Six primary factors were investigated: power (P), energy ($E_{\text{US}}$), pulsation on-time ($t_{\text{on-}}$), off-time ($t_{\text{off}}$), volume (V), and iron concentration ($\beta_{\text{Fe}}$). The primary response, z-average, was fitted to a reduced second-order regression model (Eq. (4)).

(4) z-avg.^=132.6172+0.0572⋅P−7.2850⋅E−0.1678⋅ton−0.0016⋅toff+0.8604⋅V−0.0838⋅βFe

$$-0.0238\cdot \left(t_{\text{on}}-6\right)\cdot \left(P-21.6\right)+1.0330\cdot {\left(E-2.3669\right)}^{2}-0.0759\cdot \left(t_{\text{on}}-6\right)\cdot \left(E-2.3669\right)+0.4057\cdot \left(V-6.1\right)\cdot \left(E-2.3669\right)-0.0701\cdot \left(\beta_{\text{Fe}}-5.5\right)\cdot \left(E-2.3669\right)+0.0429\cdot {\left(t_{\text{off}}-9.3333\right)}^{2}$$

**Fig. 3:**
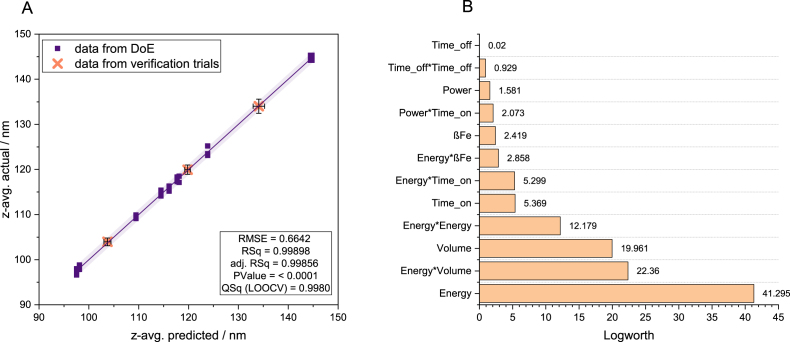
A Actual-by-predicted plot for hydrodynamic z-avg. showing design points and validation runs. B Logworth plot indicating the statistical significance of main factors and their interactions.

The model demonstrates exceptional statistical quality and predictive accuracy. The coefficient of determination (R${}^{2}$) of 0.99898 indicates that 99.9 % of the observed variance is explained by the model. To account for the number of predictors and ensure a well-specified model, the adjusted R${}^{2}$ was calculated at 0.9986, maintaining a high level of significance (p<0.0001). Crucially, the Lack-of-fit-test yielded a non-significant p-value of 0.3684, confirming that the model adequately captures the underlying data structure and that the remaining error is purely random noise. Furthermore, the model was evaluated using Leave-One-Out Cross-Validation (LOOCV) to determine its generalisability. The resulting cross-validated Q${}^{2}$ was 0.9980. The minimal discrepancy between the adjusted R${}^{2}$ and Q${}^{2}$ robustly confirms that the model is not overfitted and possesses true predictive power. Visually, the model’s performance is summarised in the actual-by-predictive plot (Fig. 3A). All design points align tightly along the identity line with a Root Mean Square Error (RSME) of 0.664 nm. To further validate the model’s robustness, three independent verification trials were conducted using parameter settings not included in the original DoE data set (Table 1).

These validation runs resulted in prediction errors of less than 1 %, squarely placing them on the identity line and confirming the model’s reliability as a precision engineering tool for process control. The relative influence of the parameters is visualised using Logworth statistics in Fig. 3B. Total energy input (E) emerges as the dominant factor (Logworth > 41), followed by energy⋅volume, volume (V) and quadratic energy ($E^{2}$) input. This interaction underscores that de-agglomeration is primarily driven by the specific energy density (E/V). While pulsation off-time and linear power settings showed negligible statistical significance within the explored experimental space, practical laboratory constrains were identified. High power settings combined with low volumes (<2 ml) can lead to a ‘dispersion’ overshoot, resulting in sample loss. Consequently, for successful laboratory scale-up, maximising $t_{\text{on}}$ and P within sage thermal limits (as discussed in Appendix C) allows for increased throughput without compromising the precise control over the final hydrodynamic diameter. These statistical findings are consistent with the physical regression coefficients of the model (Eq. (4)). Specifically, the terms identified as significant in the Logworth plot energy, volume, the quadratic term energy${}^{2}$, and the interaction terms (energy⋅volume) and (energy⋅time${}_{\text{on}}$) possess the highest weighting factors, thereby exerting the largest effect on the final particle size.

**Table 1:** Experimental validation of the predictive model.

#	P (W)	E (kJ)	$t_{\text{on-}}$ (s)	$t_{\text{off}}$ (s)	V (ml)	${\text{ß}}_{Fe}$ (mg/ml)	Predicted (nm)	Actual (nm)	Error (%)
1	15	5	10	10	2	10	103.9 ± 0.9	103.7 ± 0.7	0.22
2	30	1	5	15	5	4	134.0 ± 1.6	134.0 ± 1.2	0.06
3	20	2.5	3	5	5	1.3	120.0 ± 1.0	119.8 ± 0.6	0.13

**Fig. 4:**
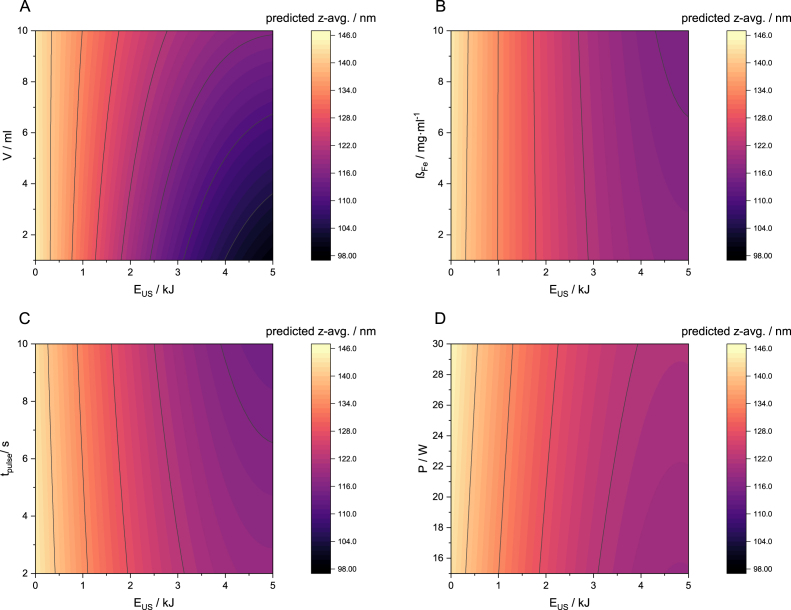
Contour plots of main factors A Contour of z-avg. depending on Volume and Energy. B Contour of z-avg. depending on Iron concentration and Energy. C Contour of z-avg. depending on on- time and Energy. D Contour of z-avg. depending on Power and Energy.

The analysis show that even though energy and volume have the greatest impact, other interactions also contribute. This is visualised in Fig. 4. Here, z-avg is shown as a function of volume and energy. Results suggest that the lowest z-avg can only be achieved by maximising the energy and minimising volume. This results in the highest energy per unit volume, which can be described as energy density. The analysis also reveals another interaction of volume and energy on z-avg especially at higher energies (>2 kJ). Here, the contour lines are more curved, when compared to the contour lines at lower energies. Apart from that, the influence of the iron concentration (Fig. 4B), is markedly less pronounced. The contour lines are nearly vertical suggesting that the impact of iron concentration in the analysed range between 1 and 10 mg/ml on z-avg. is practically negligible, especially when compared to the impact of energy and volume. This is also the case for time${}_{\text{on}}$ (Fig. 4C) and power (Fig. 4D). Consequently, a highest time${}_{\text{on}}$ and highest power can be evaluated for further scale up. The effect of all contributors is discussed as a result of the leverage plots in Figure D.5.

These findings have direct practical implications. For further scale-up, power and time${}_{\text{on}}$ can be set to their maximum values to increase throughput without negatively affecting particle size, although this requires careful temperature monitoring to prevent unwanted oxidation. Conversely, for lab-scale synthesis where precise size tuning is the primary goal, energy and volume serve as the critical control parameters.

### 3.3 Cellular loading

**Fig. 5:**
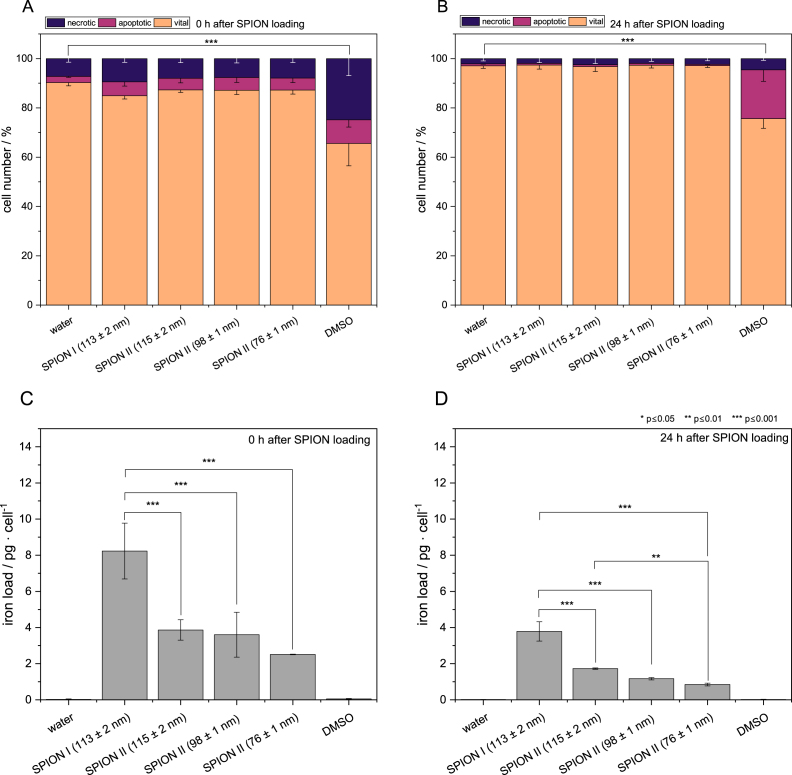
Cell viability and cellular iron load of B16-F10 cells. A Cell viability 0 h after SPION loading. B Cell viability 24 h after SPION loading. (Statistics performed by means of ANOVA and Tukey post-hoc test *** p ≤ 0.001.) C Iron load 0 h after SPION loading. D Iron load 24 h after SPION loading.

The cytotoxicity and cellular load of the SPIONs are assessed using B16-F10 cells to evaluate their performance in a biological context using sterile filtrated SPION I (z-avg of 113 nm) and SPION II (z-avg = 115, 98 and 76 nm). Particles were chosen to assure possible sterile filtration and for assessing the influence of core particle size, as z-avg of SPION I-113 and SPION II-115 is comparable (Fig. 5). Following a 24 h incubation with 75 μg/ml of SPIONs (Fig. 5A), cell viability was monitored immediately after incubation and after additional 24 h (Fig. 5B). The concentration tested, was used to maintain colloidal stability in cell culture media (Figure E.6). The viability states of the samples remain comparable to those of the negative H${}_{2}$O control, indicating minor to no detectable toxicity under the tested conditions and suggest a good biocompatibility for all SPION formulations tested. The quantification of averaged iron load in Fig. 5C and D, normalised to cell number, reveals a dual-variable dependency of nanoparticle load on both cluster and core dimensions for the tested SPION formulations. At 0 h post 24-h-incubation, the highest load can be observed for SPION I-113 nm (8.23 pg Fe/cell), followed by SPION II-115 nm (3.86 pg Fe/cell), SPION II-98 nm (3.60 pg Fe/cell), and SPION II-76 nm (2.51 pg Fe/cell). After a 24 h period, the iron content per cell remains elevated relative to the controls, but declines across all formulations (SPION I-113 nm: 3.78 pg Fe/cell; SPION II-115 nm: 1.52 pg Fe/cell; SPION II-98 nm: 1.37 pg Fe/cell; SPION II-76 nm: 0.85 pg Fe/cell). These results are consistent with the dilution of the intracellular nanoparticle load due to ongoing cell proliferation. Among the formulations examined, SPION I-113 nm demonstrates a consistent tendency to exhibit the highest Fe load, while SPION II-76 nm exhibits the lowest.

The analysis demonstrates that cluster size is not the sole predictor of high cellular SPION loading. Instead, the results indicate a dual-variable correlation on SPION load. Within the SPION II formulations, we observe that iron uptake correlates with the cluster diameter, where larger clusters exhibit higher iron loading per cell (Fig. 5D). Furthermore, when comparing SPION I and II at similar hydrodynamic sizes (approx. 115 nm), the formulation with the larger 12 nm cores (SPION I-113 nm) achieved a significantly higher iron loading (8.23 pg Fe/cell) compared to those with 8 nm cores (SPION II-115 nm: 3.86 pg Fe/cell). This highlights that the total iron mass delivered per cluster, the ‘magnetic payload’, is a critical factor for cell loading. Additionally, surface chemistry must be considered. The higher PAA content determined for SPION II (Figure A.1 B) arguably influences the SPION-cell interaction at the interface, potentially affecting the efficiency of the internalisation process or the unspecific binding on the cell surface. Even though the averaged iron content per cell does not show the true iron distribution over the analysed cell number, the effect in magnetic enrichment in Figure E.8 shows that most of the cells magnetophoretically migrate towards the applied magnetic field indicating that most cells are magnetically modified and the SPIONs are distributed over a large cell number. Taken together, these findings suggest that cellular iron loading and the resulting functional magnetic responsiveness (as demonstrated in Figure E.8) are influenced by a combination of core dimensions, cluster size, and the specific properties of the polyacrylic acid coating.

By combining the validity of our regression model for precise size tuning with these observable biological dependencies, we established a robust engineering tool for biomedical applications. This framework enables the rational design of SPION clusters to achieve predictable outcomes in cell loading, providing a reliable foundation for the optimisation of magnetic cell tracking, drug targeting, and guidance.

## 4 Conclusion

In this comprehensive study, a robust process for controlling the colloidal state and, consequently, the functional properties of polyacrylic acid-coated superparamagnetic iron oxide nanoparticles (SPIONs) for biomedical applications has been successfully developed, optimised, and validated. A systematic investigation of two distinct SPION formulations (SPION I and SPION II) was performed in order to provide a comprehensive understanding of their behaviour, ranging from fundamental material properties to their interaction with biological systems.

Our key findings demonstrate that high-energy ultrasound is a highly effective method for de-agglomerating SPION suspensions. This process significantly reduces the hydrodynamic diameter of the particles without compromising the integrity of their primary iron oxide cores, as confirmed by a combination of DLS, TEM, and magnetometry. The employment of a statistical Design of Experiments (DoE) approach resulted in the successful establishment of a predictive quantitative model. This model identified the total energy input per unit volume (E${}_{US}$/V) as the most critical parameter set governing the final particle size. This provides a crucial and reliable tool for the reproducible, scalable production of SPION dispersions with precisely tailored hydrodynamic diameters.

This study directly links the controllable physical process parameter with a predictable biological outcome. It is imperative to note that the biological evaluation demonstrated that all SPION formulations tested exhibited excellent biocompatibility for the tested concentration. The efficiency of cellular SPION loading showed a correlation with the particle’s hydrodynamic size. Specifically, cellular loading was found to strongly associated with the magnetic payload per cluster. While larger clusters lead to a higher iron content per cell, the core dimensions emerged as the key predictive parameter factor. SPIONs with 12 nm cores achieved significantly higher iron loading than their 8 nm counterparts, even when their hydrodynamic dimensions were comparable. This provides a powerful strategy for tuning nanoparticles for specific tasks, moderately aggregated particles can be engineered for high-loading applications such as cell tracking or hyperthermia, whereas fully dispersed particles could be better suited for applications requiring prolonged systemic circulation. In terms of future prospects, this work provides a comprehensive framework for the rational design and manufacturing of functional nanomaterials. The logical subsequent steps involve the translation of these findings into *in vivo* models in order to investigate how the controlled size of the particles affects bio-distribution, circulation half-life, and tumour accumulation in the case of magnetic drug targeting in combination to chemical surface modification.

Moreover, the potential exists for size-tunable SPIONs to provide greater insight into the fundamentals of *in vitro* applications, including magnetic particle spectroscopy and the tracking of magnetically labelled targets using quantum sensors.

## CRediT authorship contribution statement

**Lukas Heinen:** Writing – original draft, Visualization, Project administration, Methodology, Investigation, Formal analysis, Data curation, Conceptualization. **Pascal-Raphael Blersch:** Writing – review & editing, Investigation. **Constantin Schnell:** Writing – review & editing, Investigation. **Karsten Meyer:** Writing – review & editing, Investigation. **Roland Nagy:** Writing – review & editing. **Marcus Halik:** Writing – review & editing, Supervision. **Christina Janko:** Writing – review & editing. **Stefan Lyer:** Writing – review & editing. **Christoph Alexiou:** Writing – review & editing, Supervision, Funding acquisition. **Rainer Tietze:** Writing – review & editing, Supervision, Funding acquisition.

## Declaration of Generative AI and AI-assisted technologies in the writing process

During preparation of this work the authors used Google AI studio for improving code and text readability as well as DeepL for translation. After using these tools, the author reviewed and edited the content as needed and take full responsibility for the content of the published article.

## Declaration of competing interest

The authors declare that they have no known competing financial interests or personal relationships that could have appeared to influence the work reported in this paper.

## Data Availability

All data analysed during this study are included in this published article and its supplementary information files. Additional raw data are available from the corresponding author upon reasonable request.

## Appendix A Supplementary data

MMC S1 Supplementary Information Appendices A-E. MMC S1
