# Supplementary material for: Ultrasound-assisted size tuning of polyacrylic acid coated magnetic nanoparticle clusters for biomedical applications
Source: Ultrason Sonochem. 2026 May 13;130:107876. doi: 10.1016/j.ultsonch.2026.107876 (PMC13213284; doi:10.1016/j.ultsonch.2026.107876)
Supplement: MMC S1 — Supplementary Information Appendices A-E. [file mmc1.pdf]

Supplementary Data
Ultrasound-Assisted Size Tuning of Polyacrylic Acid Coated Magnetic Nanoparticle Clusters for Biomedical Applications

Lukas Heinen[a,d,e], Pascal-Raphael Blersch[a], Constantin Schnell[b], Karsten Meyer[b], Roland Nagy[c], Marcus Halik[d], Christina Janko[a], Stefan Lyer[a], Christoph Alexiou[a], Rainer Tietze[a]

[a] *Department of Otorhinolaryngology – Head and Neck Surgery, Section of Experimental Oncology and Nanomedicine (SEON), Else Kröner Fresenius Foundation Professorship (EKFS), Universitätsklinikum Erlangen, Erlangen, 91054, Bavaria, Germany*
[b] *Department of Chemistry and Pharmacy, Inorganic Chemistry, Friedrich-Alexander-Universität Erlangen-Nürnberg, 91058, Bavaria, Germany*
[c] *Institute of Applied Quantum Technologies, Friedrich-Alexander-Universität Erlangen-Nürnberg, 91052, Bavaria, Germany*
[d] *Organic Materials & Devices, Institute of Polymer Materials, Friedrich-Alexander-Universität Erlangen-Nürnberg, 91058, Bavaria, Germany*
[e] *corresponding author*

Appendix A. Characterisation of particles

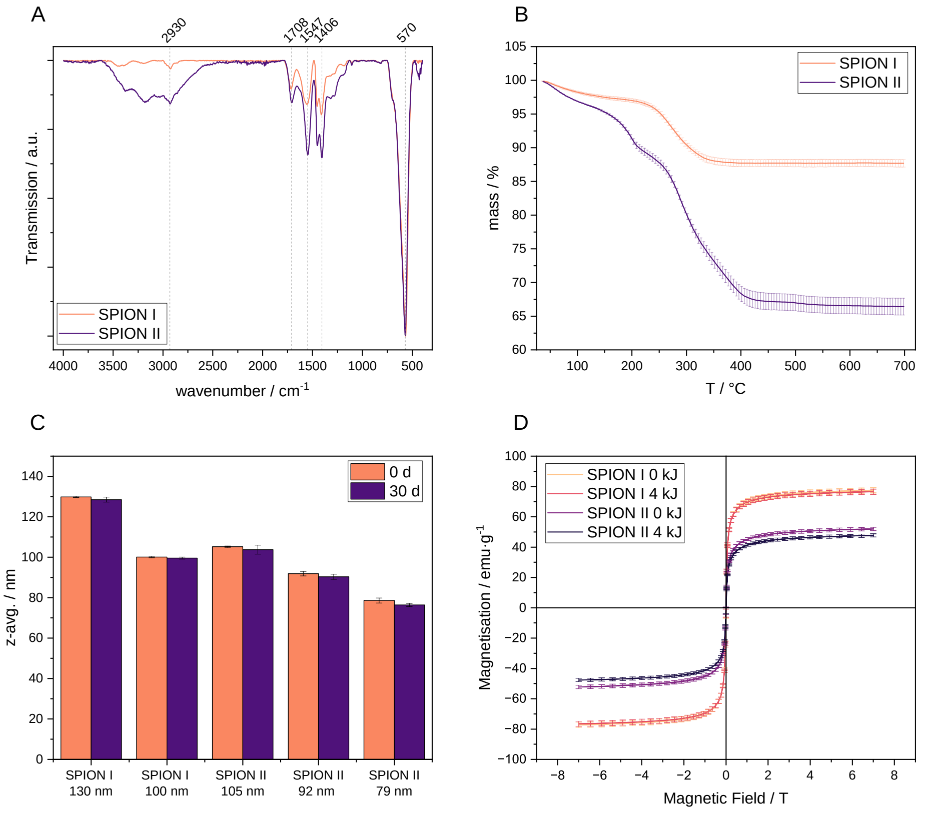

Figure A.1: **Further characterisation for SPION I and SPION II A** Infrared spectra. **B** Thermogravimetric analysis. **C** z-avg. of particle clusters immediately after sonication and 30 d after sonication. **D** Magnetisation curves for SPION I and SPION II.

Figure A.1 A compares the IR spectra of SPION I and SPION II. Attenuated total reflection spectrometry Fourier transform infrared (ATR-FTIR) spectra were recored on a Bruker ALPHA-P spectrometer (400-4000 cm^{-1}, 128 scans). Dispersions were layered and air-dried on the crystal, until the Fe-O vibration reached an absorbance of 0.3. Following atmospheric and baseline correction (OPUS software, Bruker Corporation, USA), spectra were normalised to the Fe-O vibration (540-620 cm^{-1}). The characteristic vibrational bands of the PAA shell are present in both samples, including the

C=O stretch of the carboxylic group (approx. 1705 cm^{-1}) and the prominent stretches of the deprotonated carboxylate group (1548 and 1448 cm^{-1}). Furthermore, the spectra also offer a semi-quantitative insight into the compositional differences. When comparing the relative intensity of the organic PAA bands to the inorganic Fe-O vibration (570 cm^{-1}), it is apparent that the ratio of the PAA signal to the Fe-O signal is higher for SPION II than for SPION I. This observation provides the first spectroscopic evidence that SPION II has a greater proportion of PAA relative to its iron oxide core. This is in line with the thermo-gravimetric analysis in Figure A.1 B. The relative mass loss was quantified via thermogravimetric analysis (TG209 F1 Libra, Netsch, Germany). Freeze-dried SPIONs (10 mg) were analyses in aluminium oxide crucibles under synthetic air (20 ml/min), heating from 35-700 °C at 10 °C/min. Analyses were performed in triplicate using individually synthesised batches. SPION II shows a greater mass loss relative to SPION I. PAA decomposes via dehydration, decarboxylation and chain scission above 200-250 °C [1]. The plot indicates that with the given measurement method and setup, both processes simultaneously take place. When tuned to specific hydrodynamic size, the cluster sizes remain constant for at least 30 d A.1 C).

As can be seen in Figure A.1 D, both particles show no hysteresis and therefore no remanence, exhibiting true superparamagnetic behaviour. Magnetism data of freeze dried samples, loaded within a polycarbonate gel capsule inside a plastic straw were collected on a Quantum Design MPMS-3 SQUID magnetometer. DC hysteresis loops were recorded at a constant temperature of 300 K with varying fields ranging from -7 to +7 T (sequence: 0 T → 7 T → -7 T → 7 T). Based on TGA and SQUID data, the magnetic mass normalised saturation magnetisation was estimated (Table A.1). Assuming the particles consist of magnetite and oxidise to haematite and considering this mass increase of approx. 3.4 %, the magnetisation was estimated with:

$$M_{s,\text{magnetic}} = M_{s,\text{sample}} \cdot (0.966 \cdot w_{\text{residual}})^{-1} \quad \text{(A.1)}$$

Table A.1: Estimation of magnetic mass normalised saturation magnetisation

SPION	E_{US}	M_s(sample)	R_m(TGA)	M_s(inorganic)
I	0	$77.4\ emu \cdot g^{-1}$	87.7 %	$91.4\ emu \cdot g^{-1}$
I	4	$76.6\ emu \cdot g^{-1}$	87.7 %	$90.4\ emu \cdot g^{-1}$
II	0	$52.0\ emu \cdot g^{-1}$	66.4 %	$81.1\ emu \cdot g^{-1}$
II	4	$47.7\ emu \cdot g^{-1}$	66.4 %	$74.4\ emu \cdot g^{-1}$

The resulting values for SPION I reach approx. 91 emu/g which closely approaches the theoretical bulk magnetisation of pure magnetite [2] and indicates a high degree of crystallinity likely prompted by the elevated synthesis temperature of 80 °C. In contrast, the lower saturation magnetisation of untreated SPION II at 81 emu/g is attributed to the smaller core size of approx. 8 nm where the increased surface-to-volume ratio leads to more pronounced surface spin canting and the formation of a magnetic dead layer [3, 4]. While SPION I remains magnetically stable after ultrasound treatment with only a minor decrease, SPION II exhibits a greater reduction in magnetisation from 81 to approx. 74.4 emu/g after sonication. This reduction is consistent with partial sonochemical oxidation of the magnetic cores to maghemite caused by formed radicals generated during acoustic cavitation. Smaller particles are significantly more susceptible to such surface-initiated oxidation due to their higher specific surface area and higher thermodynamic instability, whereas larger cores maintain their magnetic integrity despite extreme sonication conditions. Importantly, even after partial oxidation, SPION II remain superparamagnetic and still ensure sufficient magnetic responsiveness for biomedical applications [5].

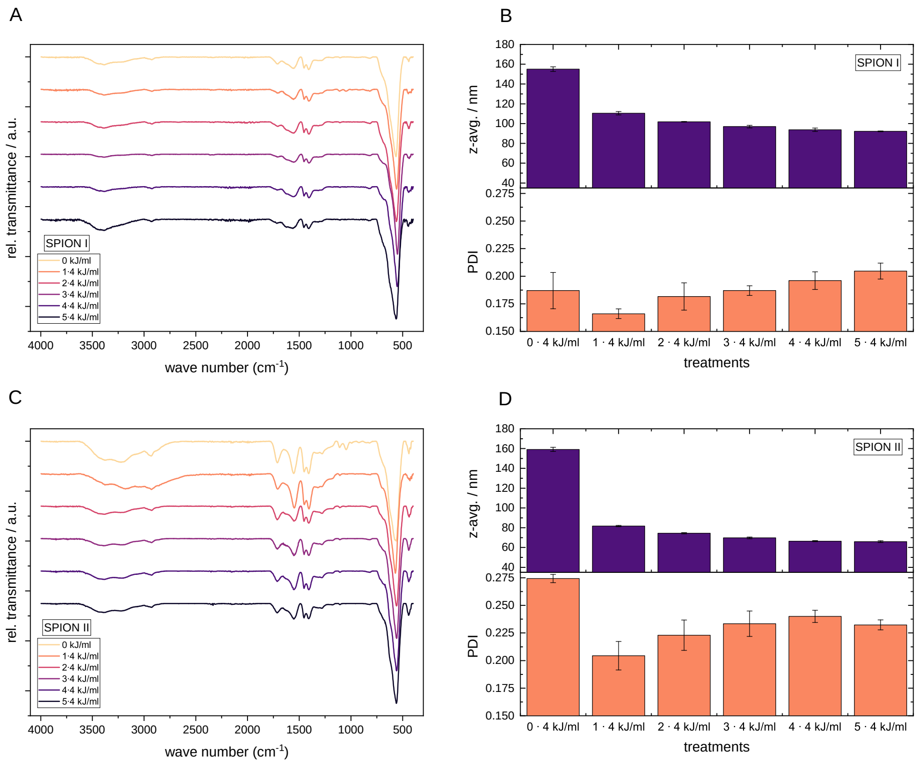

Figure A.2: **Structural and chemical robustness of SPION clusters under high-energy sonication A** Evolution of z-avg. and PDI for cumulative treatment cycles. **B** FTIR spectra of SPION samples following cumulative increasing ultrasonic energy densities.

To investigate the chemical and structural limits of the ultrasound-assisted size tuning process, both SPION formulations were subjected to cumulative energy densities far exceeding the range utilized in the experimental design (up to 20 kJ/ml, equivalent to five consecutive 4 kJ/ml treatments). Between each step, the complete water volume was exchanged using 100 kDa centrifugal filters to ensure the removal of any potentially detached polymer.

The hydrodynamic size reduction for both systems exhibits a clear plateau behaviour (Figures A.2B and D). For SPION I, the z-average converges toward a minimum primary cluster size of approximately 94 nm. In contrast, SPION II reaches a significantly lower plateau of approximately 67 nm. This difference is consistent with the smaller core size of SPION II (8 nm vs.

12 nm for SPION I), which allows for the formation of smaller stable primary clusters. In both cases, the plateau indicates that the ultrasonic energy effectively overcomes the physical adhesion between clusters but does not break the structural integrity of the primary clusters themselves.

The evolution of the Polydispersity Index (PDI) provides further insight into the de-agglomeration process. SPION II initially exhibits a very high PDI (> 0.27), which drops sharply to ≈ 0.20 after the first treatment, indicating the successful breakdown of large micrometer-scale agglomerates into a more monodisperse population. While both systems show a minor upward trend in PDI beyond 4 kJ/ml (reaching ≈ 0.21 for SPION I and ≈ 0.24 for SPION II), the values remain within the range of stable, narrowly distributed suspensions. This slight increase at extreme energy levels is likely due to over-sonication effects, such as the generation of fine fragments or minor secondary collision-induced re-agglomeration.

The FTIR spectra for both SPION I (Figure A.2A) and SPION II (Figure A.2C) demonstrate consistency across the entire range of ultrasonic energy input (0 to 20 kJ/ml). The characteristic vibrations of the PAA coating, including the C=O stretch ($\approx$ 1708 cm^{-1}), the COO$^-$ stretches (1548 and 1448 cm^{-1}), and the inorganic Fe-O vibration ($\approx$ 570 cm^{-1}), remain unchanged in position and relative intensity. This provides definitive spectroscopic proof that the ultrasound treatment is non-destructive towards the PAA coating for both formulations, even for SPION II which possesses a higher proportion of PAA relative to its iron oxide core.

Appendix B. Quantitative Analysis of Cluster Fragmentation and Magnetic Scaling

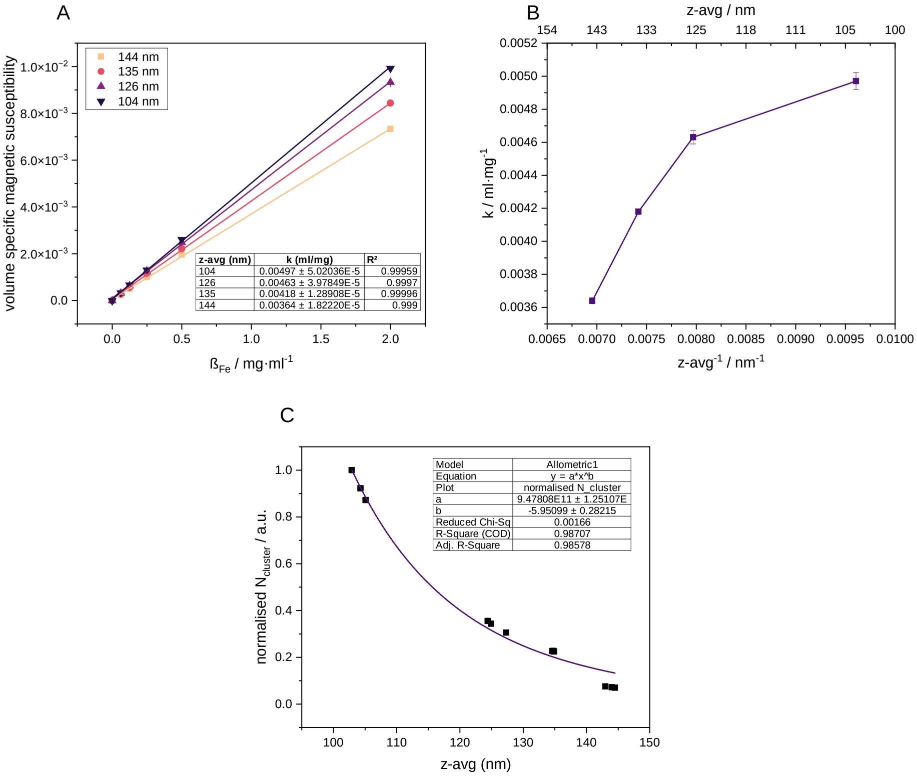

Figure B.3: **Quantitative analysis of magnetic and structural cluster properties for SPION I. A** Volume-specific magnetic susceptibility as a function of iron concentration (β_{Fe}) for different hydrodynamic diameters, showing strictly linear behavior ($R^2 > 0.999$). **B** Susceptibility slope k plotted against the inverse hydrodynamic diameter (z-avg^{-1}). **C** Allometric power-law fit of the normalized cluster number density $N_{cluster}$ as a function of z-avg ($b \approx -6$), reflecting the fragmentation kinetics and intensity-weighted nature of DLS data.

This quantitative analysis provides the physical foundation for the deagglomeration process and the resulting magnetic enhancement observed in Figure 1. As shown in Figure B.3A, the volume-specific magnetic susceptibility (χ_V) exhibits a strictly linear relationship with the iron concentration

(β_{Fe}) for all investigated cluster sizes ($R^2 > 0.999$), which confirms colloidal stability during the measurement period and definitively excludes experimental artifacts such as sedimentation or concentration-dependent volume fraction changes. For further analysis, we define the cluster-size dependent mass-specific susceptibility k as the derivative of the susceptibility with respect to the mass concentration:

$$k \equiv \frac{\mathrm{d}\chi_v}{\mathrm{d}\beta_{\mathrm{Fe}}} \tag{B.1}$$

The slopes k (ml/mg) increase significantly from 0.00364 to 0.00497 ml/mg (+36%) as clusters are fragmented from 144 nm to 104 nm, demonstrating that the clusters exhibit a higher magnetic responsiveness when distributed into smaller, independent units. To elucidate the structural origin of this enhancement, k was plotted against the inverse hydrodynamic diameter (z-avg^{-1}) in Figure B.3B. The observed trend directly supports the findings in Figure 1D of the main manuscript. Crucially, for the standardized SPION concentration of 1 mg/ml used in the main text and given the linear relationship $\chi_V = k \cdot \beta_{Fe}$, the value of k is numerically equal to χ_V. This confirms that the observed rise in susceptibility is a result of structural "de-locking" rather than a concentration effect.

The fragmentation process was further quantified using the DLS-based Malvern (Malvern Panalytical, UK) concentration tool, where the initial concentration was manually adjusted until the expected derived count rate and the measured count rate match. This was performed for each DLS measurement performed at an iron concentration of 50 µg/ml. To avoid biasing, we normalised the highest particle number to 100 %. As can be seen, the relationship between normalized cluster number density (N) and z-avg follows an allometric power-law fit $N \propto D^b$ with an exponent of $b = -5.95$ (Figure B.3C, Adj. $R^2 = 0.985$). This specific exponent is a direct consequence of the inherent intensity bias in DLS, where scattering intensity scales with the sixth power of the diameter (D^6) according to Rayleigh theory. Consequently, the estimated 15-fold increase in N reflects a massive transition from a few dominant agglomerates to a vast population of independent clusters. At constant iron mass, this redistribution therefore significantly increases the mean inter-cluster spacing, effectively reducing the probability of inter-unit magnetic coupling and flux-closure states. The observed onset of convergence in k for the smallest clusters (104 nm) suggests that the inter-unit spacing has reached a threshold where magnetic de-locking is nearly complete, while

the cluster sizes themselves approach a stable minimum dictated by the fragmentation limit of the sonication process as can be seen in Figure 1B.

Taken together, the magnetic and optical analyses further underline the success of the ultrasound-assisted de-agglomeration process and its direct impact on the functional responsiveness of the SPION clusters.

Appendix C. Temperature Profile and Energy Dissipation

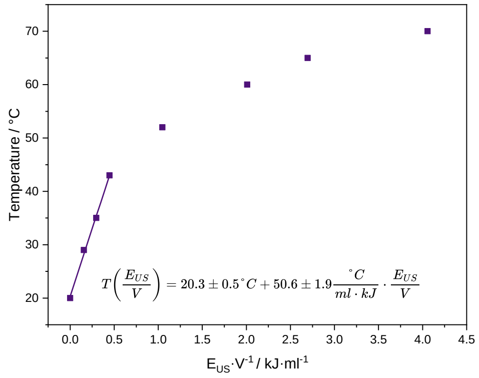

Figure C.4: Temperature profile of water as a function of the cumulative specific energy reported directly by ultrasonic generator.

To evaluate the thermal impact of the ultrasound treatment on the PAA-coated SPIONs, the bulk temperature (T) of the aqueous medium was measured as a function of the specific energy input ($E_{US} \cdot V^{-1}$) reported by the generator (Figure C.4). Energy was applied at 15 W, 10 s on- and 10 s off-time for various total times. As shown, the temperature profile exhibits a non-linear, asymptotic behaviour. This trend is expected under the non-adiabatic conditions of the experiment, where the rate of heat dissipation to the surrounding environment increases as the temperature differential between the sample vessel and the ambient air rises (following Newton's law of cooling). Crucially, the data demonstrate that even at the maximum treatment intensity of 4 kJ/ml utilized in the experimental design, the bulk temperature plateaus at approximately 70-75 °C. This temperature remains significantly below the thermal degradation threshold of the PAA coating, which exhibits a TGA onset at approximately 250 °C (see Figure A.1B). Furthermore, by remaining below the boiling point of water, the process avoids total acoustic decoupling and prevents the convective "overshoot" often observed in poorly controlled ultrasonic systems. These results confirm that the post-synthesis size tuning is a thermally safe process that preserves the chemical and colloidal integrity of the polyacrylic acid shell.

The recorded energy of the device acts as process predictor, but is not necessarily the effective energy delivered to the sample. The efficiency can roughly be estimated using the temperature plot. Considering only the linear

part, the used volume, the heat capacity of water ($4.18\ J/gK$) and the non-isolated set up the efficiency η can be defined as the ratio of thermal energy over the generator reported ultrasound energy.

$$\eta = \frac{Q}{E_{US}} = \frac{m \cdot c_p \cdot \Delta T}{E_{US}} \tag{C.1}$$

A temperature increase from 20 to 45 °C upon 500 J sonication yields an efficiency of approx. 21 % and falls below typical electrical-to-acoustic conversion efficiencies reported in the literature of 60–70 %. This is expected, as the present metric captures the full energy cascade from the generator's nominal output to heat deposited in the sample, rather than solely the electrical-to-acoustic conversion at the transducer. Dissipations occur during the processes of energy conversion, resulting in acoustic powers that are significantly lower than the respective nominal power that the equipment requires from the energy source. Additional losses are attributable to the small sample volume of 1 mL, which increases the surface-to-volume ratio and therefore convective heat loss to the environment, as well as the non-isolated experimental setup. [6, 7, 8]

Appendix D. Scaling ultrasound treatment

With the given model validity in Figure 3 and Table 1, the leverage plots (Figure D.5) visualise the impact of each model term from Equation 3. The analysis unequivocally identifies Energy and Volume as the most influential parameters. Energy exhibits a strong linear effect ($P<0.0001$). Critically, the highly significant positive quadratic energy term ($P<0.0001$), reveals a pronounced convex curvature in response. This indicates that although initial increase in energy are highly effective at reducing z-avg., the effect exhibits diminishing returns at higher energy levels, which is consistent with the observation in the small scaled experiments in Figure 1 B. Volume shows a strong positive linear effect ($P<0.0001$), suggesting that sonication is less efficient in larger volumes. The dominant impact of both, energy and volume can be explained by the classical principle of energy density. For a fixed total energy input, a larger volume results in lower energy density, reducing the efficiency of cavitation-induced de-agglomeration an thus leading to a larger final particle size. The refined model reveals that on-time is a statistically significant, albeit secondary, factor ($P<0.0001$). Its effect, while much smaller in magnitude than energy or volume, provides another lever for process control. The model also confirms the importance of on- time ($P<0.0001$) and iron concentration ($P=0.0038$) as minor but real contributors. A key outcome of this expanded design was the screening-out of off time as an influential parameter. Both its linear ($P=0.9547$) and quadratic ($P=0.1178$) terms were found to be insignificant. This is a valuable finding, as it simplifies the process by demonstrating that the resting period between sonication, and therefore temperature especially at higher energy inputs, does not impact the final z-avg. The model's high accuracy is also enabled by the capture of key interactions. The energy·volume interaction ($P<0.0001$). remains the most critical interaction. The significance of the power· on-time interaction ($P=0.0084$) is particularly insightful. It indicates that the effect of power is not constant but depends on the on-time setting. This synergistic relationship explains why power is a relevant factor despite its smaller main effect. Other interactions involving energy·(on-time, iron concentration) further refine the model, accounting for more subtle complexities in the system. Experimental designs and results can be taken from Table D.2 and Table D.3.

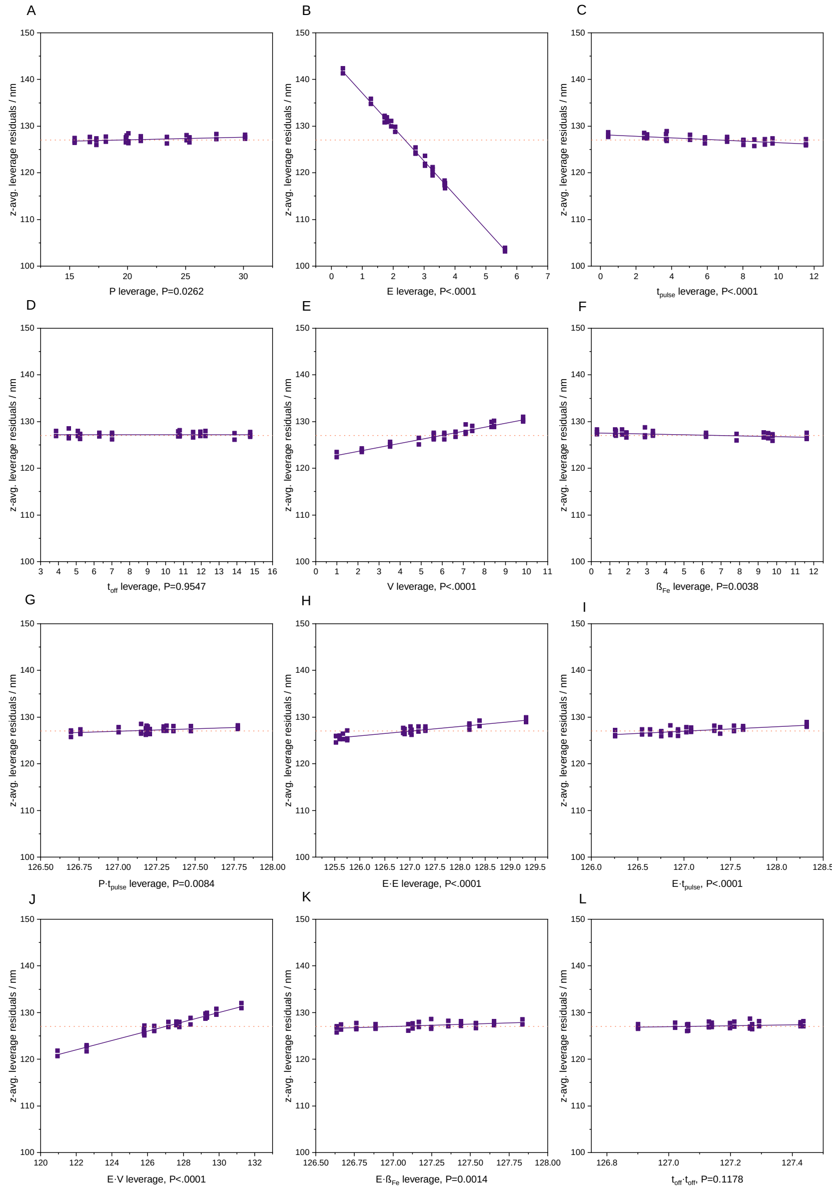

Figure D.5: **Graphical summary of the multiple regression model for z-avg.** The actual by predicted plot (top) demonstrates the model's predictive accuracy. Other leverage plots show the impact of the respective factors or factor combination on z-avg. A high slope indicates a strong impact.

Table D.2: Experimental design and measured values for z-avg. and PDI.

#	P (W)	E (kJ)	$t_{\text{on- time}}$ (s)	$t_{\text{off time}}$ (s)	V (ml)	$ß_{Fe}$ (mg/ml)	z-avg (nm)	PDI
1	30	0.0	2	10	10	10	145.3	0.168
1	30	0.0	2	10	10	10	144.2	0.168
1	30	0.0	2	10	10	10	144.2	0.171
2	30	0.0	10	5	1	5	145.3	0.168
2	30	0.0	10	5	1	5	144.2	0.168
2	30	0.0	10	5	1	5	144.2	0.171
3	15	5.0	6	5	1	10	97.97	0.199
3	15	5.0	6	5	1	10	97.62	0.19
3	15	5.0	6	5	1	10	96.58	0.209
4	23	5.0	10	15	10	10	114.3	0.173
4	23	5.0	10	15	10	10	115.4	0.166
4	23	5.0	10	15	10	10	114.1	0.18
5	30	0.0	6	15	10	1	145.3	0.168
5	30	0.0	6	15	10	1	144.2	0.168
5	30	0.0	6	15	10	1	144.2	0.171
6	23	0.0	2	5	1	1	145.3	0.168
6	23	0.0	2	5	1	1	144.2	0.168
6	23	0.0	2	5	1	1	144.2	0.171
7	30	5.0	10	5	10	1	116.3	0.199
7	30	5.0	10	5	10	1	116.2	0.161
7	30	5.0	10	5	10	1	115.2	0.179
8	30	5.0	2	5	5.5	10	109.5	0.175
8	30	5.0	2	5	5.5	10	109.9	0.189
8	30	5.0	2	5	5.5	10	109.1	0.185
9	15	2.5	2	5	10	1	125.2	0.196
9	15	2.5	2	5	10	1	123.1	0.177
9	15	2.5	2	5	10	1	123.5	0.163
10	15	5.0	10	10	1	1	98.24	0.207
10	15	5.0	10	10	1	1	98.81	0.206
10	15	5.0	10	10	1	1	97.83	0.187
11	15	0.0	10	5	10	10	145.3	0.168
11	15	0.0	10	5	10	10	144.2	0.168
11	15	0.0	10	5	10	10	144.2	0.171

Table D.3: Experimental design and measured values for z-avg. and PDI continued. °denotes not applicable combination due to liquid overshoot.

#	P (W)	E (kJ)	$t_{on\text{- time}}$ (s)	$t_{off\ time}$ (s)	V (ml)	$ß_{Fe}$ (mg/ml)	z-avg (nm)	PDI
12	30	2.5	10	15	1	10	°	°
12	30	2.5	10	15	1	10	°	°
12	30	2.5	10	15	1	10	°	°
13	30	5.0	2	15	1	1	°	°
13	30	5.0	2	15	1	1	°	°
13	30	5.0	2	15	1	1	°	°
14	15	0.0	10	15	5.5	1	145.3	0.168
14	15	0.0	10	15	5.5	1	144.2	0.168
14	15	0.0	10	15	5.5	1	144.2	0.171
15	23	2.5	6	10	5.5	5.5	118.5	0.163
15	23	2.5	6	10	5.5	5.5	118.5	0.172
15	23	2.5	6	10	5.5	5.5	117.1	0.181
16	15	5.0	2	15	10	5.5	117.3	0.174
16	15	5.0	2	15	10	5.5	118.3	0.157
16	15	5.0	2	15	10	5.5	117.6	0.178
17	15	0.0	2	15	1	10	145.3	0.168
17	15	0.0	2	15	1	10	144.2	0.168
17	15	0.0	2	15	1	10	144.2	0.171

Appendix E. Bio characterisation

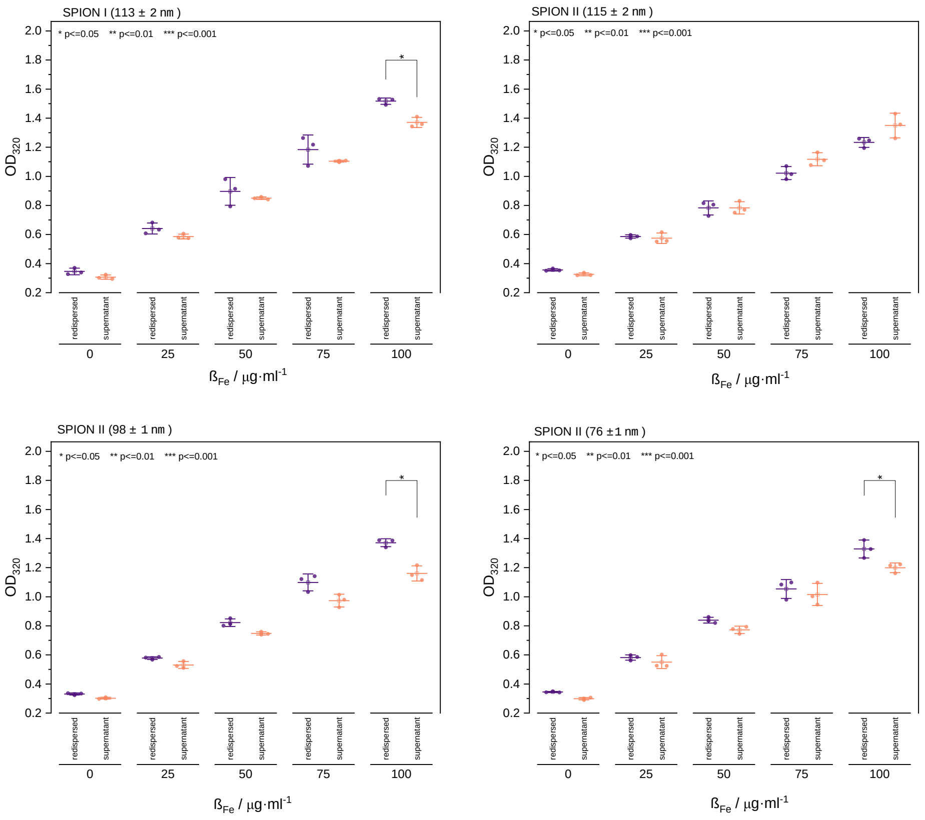

Figure E.6: Concentration dependent colloidal stability in cell culture medium of SPION I and SPION II

Prior to biological tests, colloidal stability of SPIONs in cell culture medium was assessed by means of optical density measurements. SPIONs at different concentration were incubated with cell culture medium for 24 h at 37 °C in a total volume of 200 µl in a 96-well plate. Following incubation, 100 µl supernatant was carefully removed and transferred to another well. The remaining 100 µl were then re-dispersed and the optical density at 320 nm (OD_{320}) was measured using a micro plate reader (SpectraMax iD3, Molecular Devices, Germany). Samples are not colloidally stable if there is a significant difference between supernatant and re-dispersed sample. Figure

E.6 shows that a iron concentration of 75 µg/ml remains colloidally stable for all SPIONs tested and serve as a good loading concentration for comparing cellular loading.

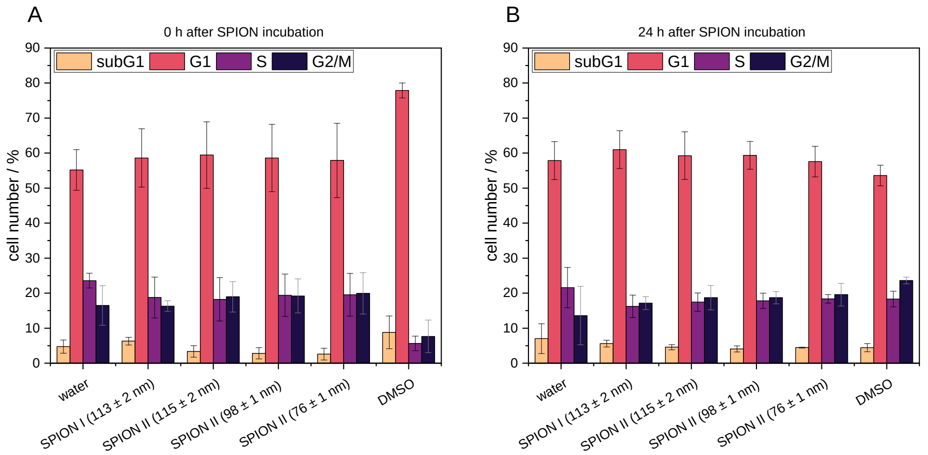

Figure E.7: **Cell cycle analysis after SPION exposure** Analysis of cell cycle phases (sub-G1, G1, S, G2/M) via Propidium Iodide (PI) staining at 0 h (**A**) and 24 h (**B**) post-incubation.

Following 24 h of SPION exposure, cells were washed with PBS, harvested, and fixed in ice-cold 70% ethanol. Fixed cells were stored at –20 °C for at least 2 h. Prior to analysis, cells were washed with PBS and stained with PI (50 µg/ml) and RNase A (100 µg/ml) in PBS for 30 min at room temperature in the dark. DNA content was measured with Gallios flow cytometer (Beckman Coulter, Inc, US), and cell cycle distribution (G_1, S, G_2/M) was analysed using Kaluza software (version 2.1). Subsequent cell cycle analysis corroborates the ongoing proliferation, demonstrating no significant alteration in the distribution of cells across sub-G1, G1, S, and G2/M phases subsequent to SPION loading. The positive control group (DMSO) shows significant changes, with an increase in the G1 population ($p < 0.001$) with a corresponding decrease in the S-phase fraction ($p = 0.003$).

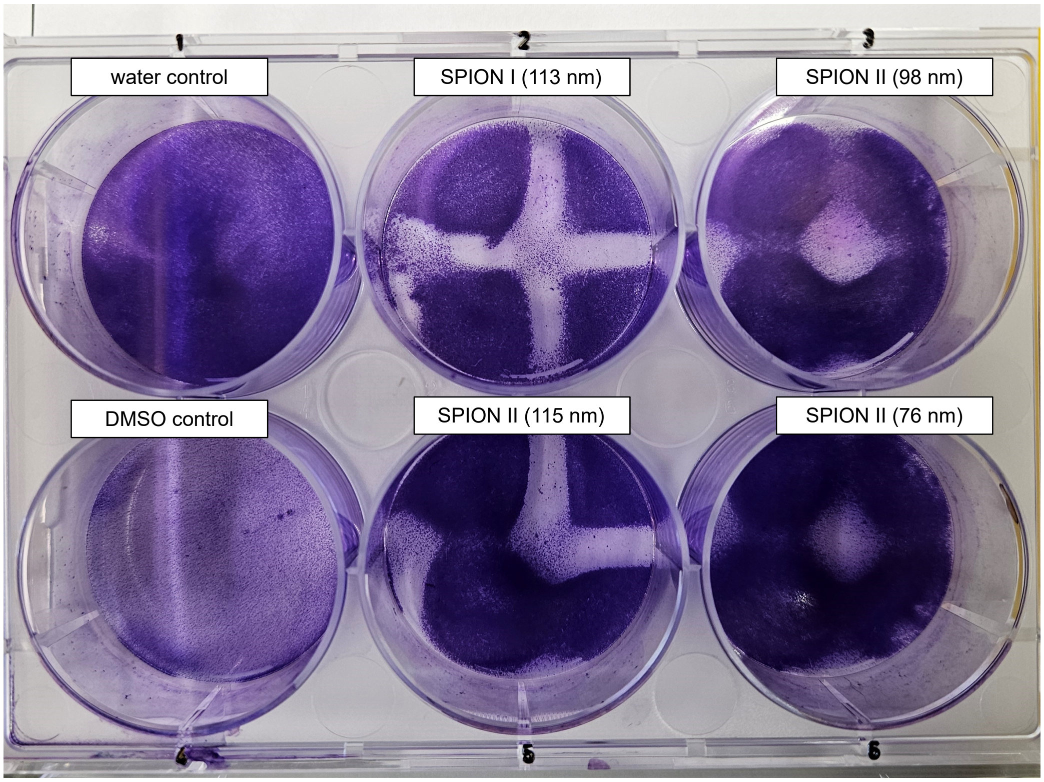

Figure E.8: **Magnetic manipulation of SPION-loaded cells** (Water and DMSO, left column) exhibit a homogeneous cell distribution. In contrast, cells incubated with SPION I and SPION II (middle and right columns) display distinct accumulation patterns mirroring the magnetic field.

The extent of magnetic responsiveness is demonstrated by applying a crystal violet stain to the sample following exposure of SPION-loaded cells to 0.5 tesla magnets for a period of 60 s (Figure E.8). A discernible separation can already be observed for the lower time period of 10 s (not shown).

The visual inspection of the culture plates reveals a stark contrast between the control groups and the particle-treated cells. The water and DMSO controls exhibit a uniform staining across the entire well surface, indication a homogeneous cell layer unaffected by the external magnetic field. In contrast, the wells containing cells incubated with SPION I and SPION II display distinct, non-homogeneous staining patterns. The cells have migrated and accumulated in specific regions corresponding to the highest magnetic field of the underlying magnets. This formation of dense cell clusters (in-

dicated by dark violet regions) and cell-depleted zones (clear region) serves as a macroscopic proof of successful magnetic labelling. Crucially, this magnetic guidance effect is clearly discernible even after a short exposure time. This rapid response indicates that the SPIONs are successfully internalised or firmly attached to the cell surface. Furthermore, it confirms that the particles retain their magnetic properties within the biological environment, generating a total magnetic moment per cell sufficiently high to overcome both thermal fluctuations and fluid resistance. Even though the averaged iron content per cell does not show the true iron distribution over the analysed cell number, the image shows that most of the cells magnetophoretically migrate towards the applied magnetic field, indicating a SPION load across a large number of cells.

References

[1] I. C. McNeill, S. Sadeghi, Thermal stability and degradation mechanisms of poly(acrylic acid) and its salts: Part 1—poly(acrylic acid), Polymer Degradation and Stability 29 (2) (1990) 233–246. doi:10.1016/0141-3910(90)90034-5.

[2] B. D. Cullity, C. D. Graham, Introduction to magnetic materials (Feb 2008). doi:10.1002/9780470386323.

[3] Z. Shaterabadi, G. Nabiyouni, G. F. Goya, M. Soleymani, The effect of the magnetically dead layer on the magnetization and the magnetic anisotropy of the dextran-coated magnetite nanoparticles, Applied Physics A 128 (8) (Jul 2022). doi:10.1007/s00339-022-05675-x.

[4] M. P. Morales, S. Veintemillas-Verdaguer, M. I. Montero, C. J. Serna, A. Roig, L. Casas, B. Martínez, F. Sandiumenge, Surface and internal spin canting in γ-fe2o3 nanoparticles, Chemistry of Materials 11 (11) (1999) 3058–3064. doi:10.1021/cm991018f.

[5] H. Unterweger, C. Janko, M. Schwarz, L. Dézsi, R. Urbanics, J. Matuszak, E. Őrfi, T. Fülöp, T. Bäuerle, J. Szebeni, C. Journé, A. R. Boccaccini, C. Alexiou, S. Lyer, I. Cicha, Non-immunogenic dextran-coated superparamagnetic iron oxide nanoparticles: a biocompatible, size-tunable contrast agent for magnetic resonance imaging, International journal of nanomedicine 12 (2017) 5223–5238. doi:10.2147/IJN.S138108.

[6] R. A. Al-Juboori, T. Yusaf, L. Bowtell, V. Aravinthan, Energy characterisation of ultrasonic systems for industrial processes, Ultrasonics 57 (2015) 18–30. doi:10.1016/j.ultras.2014.10.003.

[7] L. P. Ferraz, E. K. Silva, Unraveling the thermal effects of high-intensity ultrasound: A practical guide to acoustic power determination and heat management, ACS Omega 10 (20) (2025) 20277–20285. doi:10.1021/acsomega.4c11498.

[8] Q. Zaib, F. Ahmad, Experimental modeling to optimize the sonication energy in water, Measurement 163 (2020) 108039. doi:10.1016/j.measurement.2020.108039.
